# Transcriptome profile in bursa of Fabricius reveals potential mode for stress-influenced immune function in chicken stress model

**DOI:** 10.1186/s12864-018-5333-2

**Published:** 2018-12-13

**Authors:** Yanhua Zhang, Yanting Zhou, Guirong Sun, Kui Li, Zhuanjian Li, Aru Su, Xiaojun Liu, Guoxi Li, Ruirui Jiang, Ruili Han, Yadong Tian, Xiangtao Kang, Fengbin Yan

**Affiliations:** College of Animal Science and Veterinary Medicine, Henan Agricultural University, Zhengzhou, 450002 China

**Keywords:** Stress, Immune function, Corticosterone hormone, Bursa of Fabricius, Chicken

## Abstract

**Background:**

The molecular mechanisms underlying stress-influenced immune function of chicken (*Gallus Gallus*) are not clear. The stress models can be established effectively by feeding chickens corticosterone (CORT) hormone. The bursa of Fabricius is a unique central immune organ of birds. RNA-Seq technology was used to investigate differences in the expression profiles of immune-related genes and associated pathways in the bursa of Fabricius to clarify molecular mechanisms. The aim of this study was to broaden the understanding of the stress-influenced immune function in chickens.

**Results:**

Differentially expressed genes (DEGs) in the bursa of Fabricius between experimental group (basal diet with added CORT 30 mg/kg; C_B group) and control group (basal diet; B_B group) were identified by using RNA-seq technology. In total, we found 1434 significant DEGs (SDEGs), which included 199 upregulated and 1235 downregulated genes in the C_B group compared with the B_B group. The immune system process GO term was the top significantly GO term, including *MYD88*, *TLR4*, *IL15*, *VEGFA* gene and so on. The cytokine-cytokine receptor interaction pathway and the Toll-like receptor signaling pathway were the key pathways affected by stress. The protein-protein interaction (PPI) analysis of the SDEGs showed that *VEGFA*, *MyD88* and *IL15* were hub genes and module analysis showed that *MYD88*, *TLR4* and *VEGFA* play important roles in response to stress.

**Conclusion:**

This study showed that the *VEGFA* and *ILs* (such as *IL15*) via the cytokine-cytokine receptor interaction pathway, *MYD88* and *TLR4* via the Toll-like receptor signaling pathway may play important roles in the regulation of immune function under stress condition with CORT administration. The results of this study provide a reference for further studies of the molecular mechanisms of stress-influenced immune function.

**Electronic supplementary material:**

The online version of this article (10.1186/s12864-018-5333-2) contains supplementary material, which is available to authorized users.

## Background

Stress is a physiological manifestation of the body’s defence against adverse environmental effects. It is nonspecific responses of the body to adapt to the environment and maintain homeostatic equilibrium balance [1]. With the rapid development of the modern poultry industry and highly intensive production management, more and more stress factors have appeared in the production process, such as damp-heat, congestion, transportation, conventional vaccination, rapid cultivation of high production breeds and so on. Moderate stress can promote the immunity of the body, but excessive stress not only affects the growth, production and reproduction performance but also affects the immune system of animals, causes immune suppression, diseases and even death [2]. Stress can increase the level or secretion of adrenaline; increase the blood pressure; increase the level of corticosteroids in the plasma; contribute to the degeneration of the immune organs such as thymus, bursa of Fabricius, spleen and lymphatic tissue [3]; change the number of leukocytes [4], thereby affecting the presence of the eosinophilic and heterophilic leukocytes [5]; cause T lymphocyte injury and macrophage inhibition; and increase the catabolism of Immunoglobulin G [6], thereby resulting in a decrease in immune response and a high susceptibility to epidemics. The influence of stress on poultry production is becoming more and more serious, which has brought huge economic losses to the modern poultry industry. For a long time, researchers and poultry producers have done researches and practical works on the prevention and control of production stress [7–10], but they have not fundamentally solved this problem.

Many stress factors are present during the intensive production of poultry, and single stress factors are not representative. A stress model, therefore, is an important means for studying the stress problem. It has an irreplaceable role in the study of the mechanism of stress and the response to stress, the avoidance of stress, and in research on the drugs that interfere with stress. Corticosterone, which is secreted by the adrenal cortex and is the main glucocorticoid of birds, is involved in the stress response of the body; regulates the metabolism of carbohydrate [11], lipid [12] and protein [13, 14]; maintains the normal activities of various tissues and organs; has roles in anti-inflammatory and inhibition of immune function; and has reduced humoral and cellular immune functions [11, 15]. The establishment of the chicken stress model with exogenous CORT is theoretically based and is feasible [16].

Researches on the effect of various stress on chicken immune function in local or commercial breeds is mainly evaluated by the changes in relative lymphoid organ weight [17], partial immune related indices [18], cytokines levels [19], immune cell apoptosis [20, 21] and so on. RNA-Seq has been used to analysis the transcriptomes of the different cells and tissues from various animals [22–24]. Applying transcriptome technology to study specific stress were paid more attention to growth and development of chicken [25, 26]. Studies related to poultry stress affecting immune organ function using RNA-Seq technology has been not reported. The bursa of Fabricius is the unique central immune organ of birds and is the location of B lymphocyte differentiation and maturation. Fully differentiated B lymphocytes migrate from the bursa of Fabricius to peripheral lymphoid organs to colonize, reproduce and perform important immune functions. B lymphocytes can be converted into plasma cells and produce antibodies that participate in humoral immunity. B lymphocytes are the only cells in the body that produce antibodies that play a central role in the immune response process [27].

Therefore, to analysis molecular regulation mechanisms of the stress influencing immune function, we administered CORT in an experimental diet that included 30 mg CORT/kg basal diet to build a stress model according to previous studies [16]. Then, the bursas of Fabricius from the chicken models were analyzed via transcriptome profiling by RNA-seq technology to deeply explore genes or pathways involved in the stress response. Verifying the function of these key genes involved in the immunoregulation can lay a foundation for the analysis of molecular regulation mechanisms of the stress influencing immune function.

## Results

### Illumina sequencing and analysis of mRNA expression in the bursa of Fabricius

Six libraries B_B_1, B_B_2 and B_B_3 from the control group and C_B_1, C_B_2 and C_B_3 from the experimental group were sequenced on an Illumina HiSeq platform, with 343 million reads in total being generated, of which 95.97% (329 million) passed the filter for clean reads. Of these, 85.22 to 86.94% clean reads were aligned to the chicken genome (*Gallus gallus* 4.0), the multiple mapped reads ranged from 1.84 to 1.90%, and the uniquely mapped reads ranged from 83.32 to 85.04%. Among these mapped reads, 68.1 to 72.0% were mapped in annotated exons, 19.3 to 22.9% were mapped in the intergenic regions, and 8.3 to 9.0% were mapped in introns. Most of reads were mapped in the same coding regions as observed in the previous study [22]. The GC contents of the clean reads was 48.33 to 49.7% (Additional file 1: Table S1). To test the reliability of the samples and whether the sample selection was reasonable, we performed a correlation analysis of the 6 samples. The results (Additional file 2: Fig. S1) showed that the intra-group correlation coefficient R^2^ was approximately 0.99, which was higher than the correlation coefficient R^2^ of approximately 0.93 between the groups (R^2^ > 0.92 indicated that the experimentation was relatively reasonable).

### Analysis of gene expression levels in the bursa of Fabricius and DEGs introduced by CORT added to feed

Compared the sequencing results of two groups with the reference gene group, 19,254 genes were identified, of which 18,684 and 18,814 genes were identified in the experimental group and control group, respectively. Further analysis of the transcriptome data revealed that there were 440 and 570 genes specifically expressed in the experimental and control groups, respectively (Additional file 3: Fig. S2). Among the 19,254 genes, 15,832 had annotated information, whereas 3422 were unknown genes. The most highly expressed gene in the C_B group was cathelicidin-B1-like (*CATHB1*), and the gene was more highly expressed than in the B_B group although not significant difference. The highest expressed genes in the B_B group were the *β*-2-microglobulin (*B2M*, padj = 1.21E-86, log_2_FC = − 1.08) and immunoglobulin lambda-like polypeptide 1 (*IGLL1*, padj = 1.21E-51, log_2_FC = − 1.18), which were associated with immune response and were significantly upregulated expressed than in the C_B group (Additional file 4: Table S2).

**Fig. 1:**
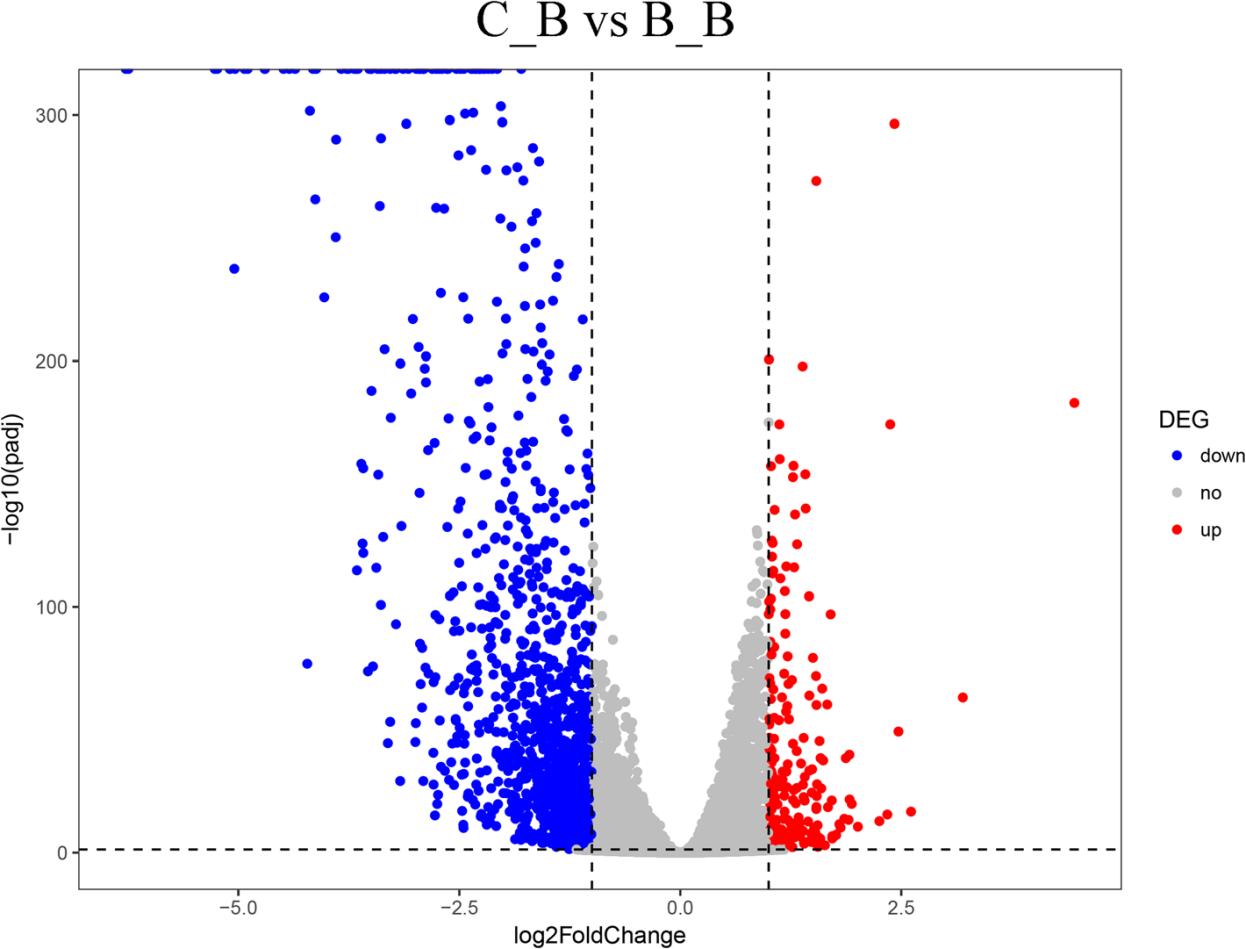
Comparison of SDEGs between the two groups. Scatter plot showing the correlation of gene abundance. SDEGs are shown as red (up) or blue (down) dots and gray dots indicate genes that lack of significantly difference

**Fig. 2:**
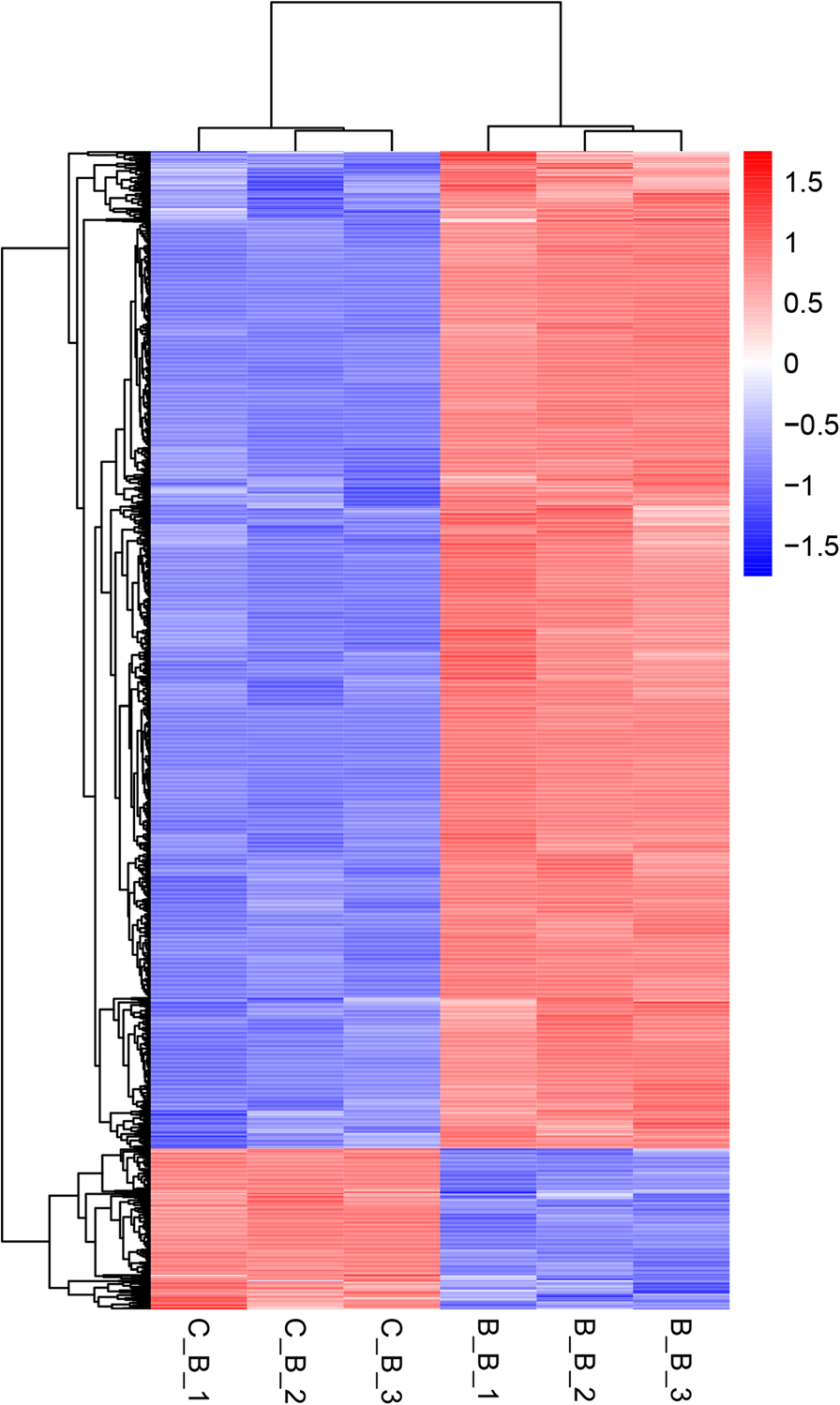
Cluster analysis of SDEGs by the FPKM value. The X-axis indicates the samples in the different groups. The three samples on the left are from the C_B group, and the three samples on the right are from the B_B group. The Y-axis is the gene cluster across the C_B and B_B group. Red indicates the high expression genes, and blue indicates the low expression genes by the value of log_10_(FPKM + 1)

In total, we found 1434 SDEGs, which included 199 upregulated and 1235 downregulated genes in the C_B group compared with the B_B group (Fig. 1; Additional file 5: Table S3). Herein, we marked the top 20 up- and downregulated genes between the C_B and B_B group according to the log_2_FC in Additional file 5: Table S3. Significantly downregulated genes included the radical S-adenosyl methionine domain-containing 2 (*RSAD2*); chemokine (C-C motif) ligand 19 (*CCL19*); chemokine-like ligand 1 precursor (*CCL4*); immunoresponsive 1 homolog (*IRG1*); *IL4I1* and *CCLI10*. Significantly upregulated genes included the heterogeneous nuclear ribonucleoprotein K (*HNRPK*); Gallinacin-2 (*GAL2*) and *CACNB4*. The heat map of cluster analysis of SDEGs showed that the gene expression patterns were similar intra-group, while were different between groups (Fig. 2).

### Gene ontology (GO) enrichment analysis

**Fig. 3:**
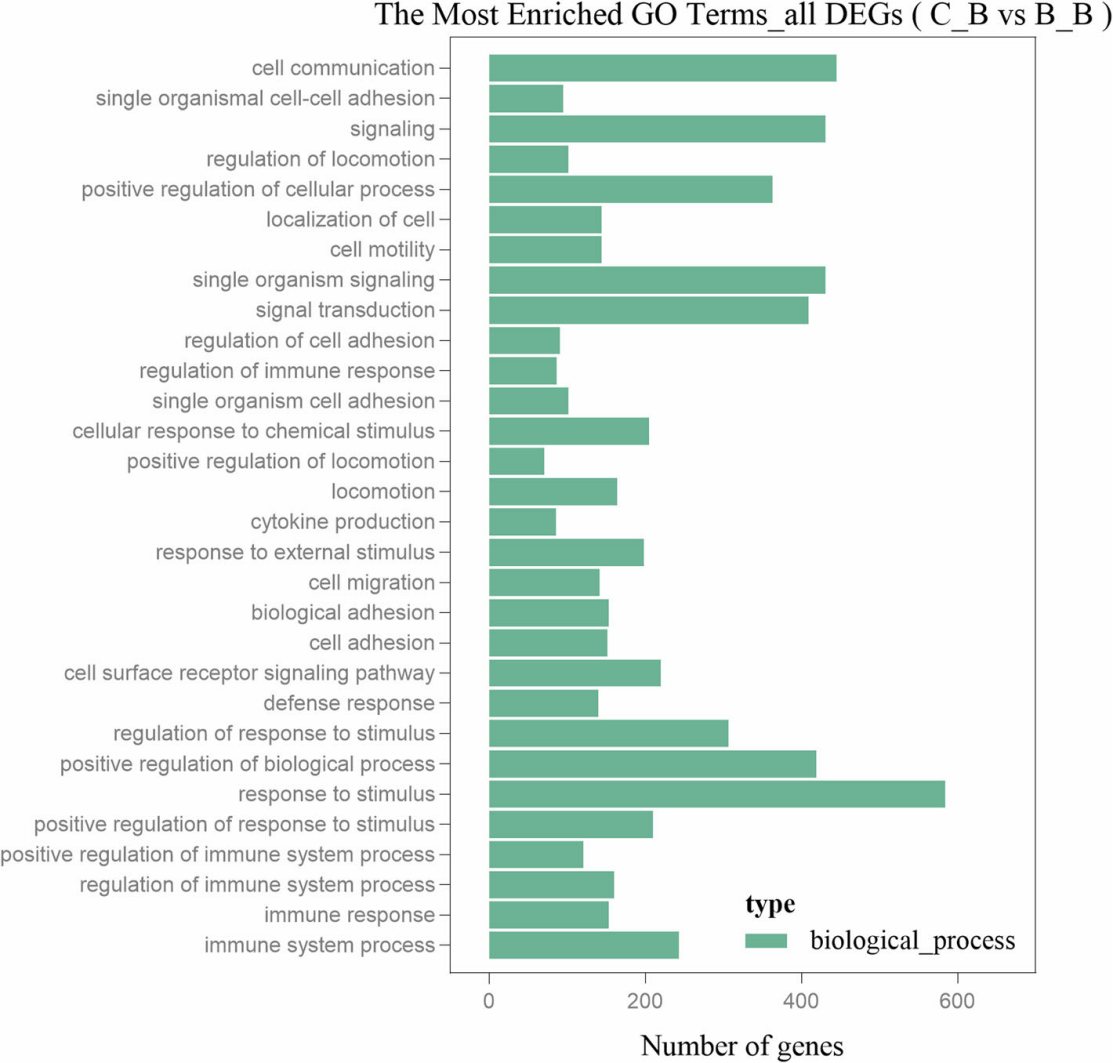
The top 30 significantly enriched GO terms of the SDEGs. The X-axis indicates the number of DEGs for each GO terms; the y-axis corresponds to the GO terms

Among the 1434 SDEGs, 1269 SDEGs of the C_B group/B_B group were enriched for 1052 GO terms (Additional file 6: Table S4). The top 30 most significantly enriched GO terms are shown in Fig. 3 (For details see Additional file 6: Table S4), which were all biological process terms (including immune system process, immune response, regulation of immune system process and so on). The immune system process was the most significantly enriched GO term. Totally, 243 SDEGs were enriched in the immune system process, including *VEGFA*, *ILs* (such as *IL-1 β*, *IL8*, *IL7*, *IL18*), *THBS1*, *TLR4*, *TLR3*, *MYD88*, *ITGB3* and other genes, which may play important roles in the process of stress affecting immune function.

### The Kyoto encyclopedia of genes and genomes (KEGG) pathway enrichment analysis

**Fig. 4:**
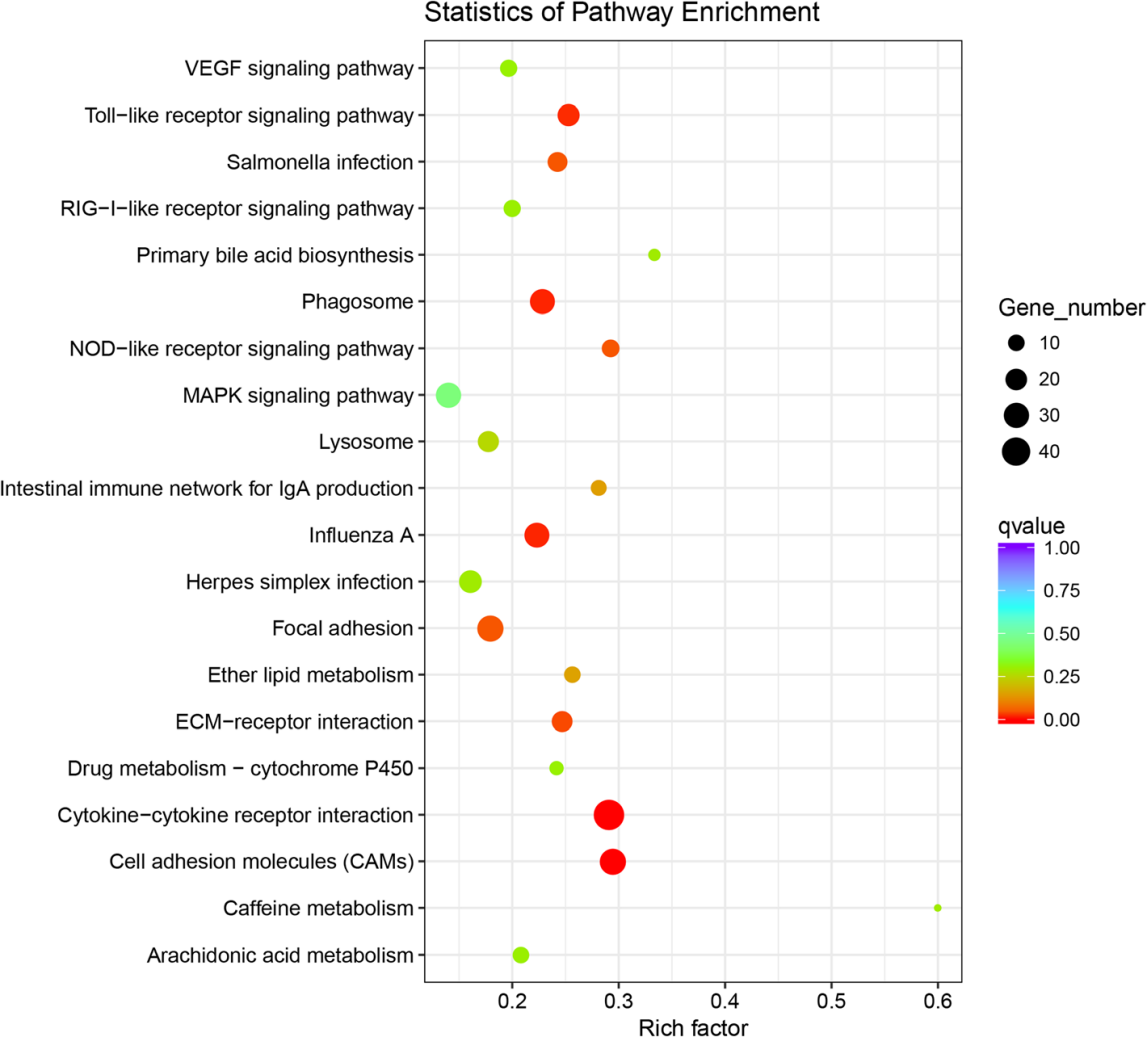
The top 20 significantly enriched KEGG pathway of the SDEGs. The x-axis shows the rich factor; the y-axis corresponds to the KEGG pathways. The dot color represents the q-value, and the dot size represents the number of SDEGs enriched in the reference pathway

KEGG pathway enrichment was used to identify the significantly biochemical metabolic pathways and signal transduction pathways modulated by CORT. The SDEGs of the C_B group/B_B group were annotated into 127 KEGG pathways. The top 20 enriched pathways of the SDEGs for the C_B and B_B group are shown in Fig. 4 (For details see Additional file 7: Table S5). In total, 9 KEGG pathways were significantly enriched, including the cytokine-cytokine receptor interaction pathway, the cell adhesion molecules pathway, the phagosome pathway, the influenza A pathway, the Toll-like receptor signaling pathway, the ECM-receptor interaction pathway, the NOD-like receptor signaling pathway, the focal adhesion pathway and the salmonella infection pathway.

The cytokine-cytokine receptor interaction pathway was the most significantly enriched pathway. We found that 48 SDEGs were enriched in the cytokine-cytokine receptor interaction pathway for the C_B vs B_B comparisons, including vascular endothelial growth factor (*VEGFA)*, *IL8*, *IL18*, *IL7*, *IL-1 β*, *IL15*, *CCL4*, *IL1R2 and so on*. The Pearson’s correlation coefficient was 0.92 between the expression of the receptor genes and ligand genes in the cytokine-cytokine receptor interaction pathway (Additional file 8: Table S6). Interestingly, we found that the 48 genes enriched in the cytokine-cytokine receptor interaction pathway were all downregulated. Similarly, the 21 genes (including myeloid differentiation primary response protein 88 (*MYD88)*, *IL-1 β*, *TLR4*, *k60*, *IL8* and so on) were enriched in the Toll-like receptor signaling pathway and all but one gene (*TLR1LA*) were also downregulated in C_B group.

### The protein-protein interaction (PPI) network analysis

**Fig. 5:**
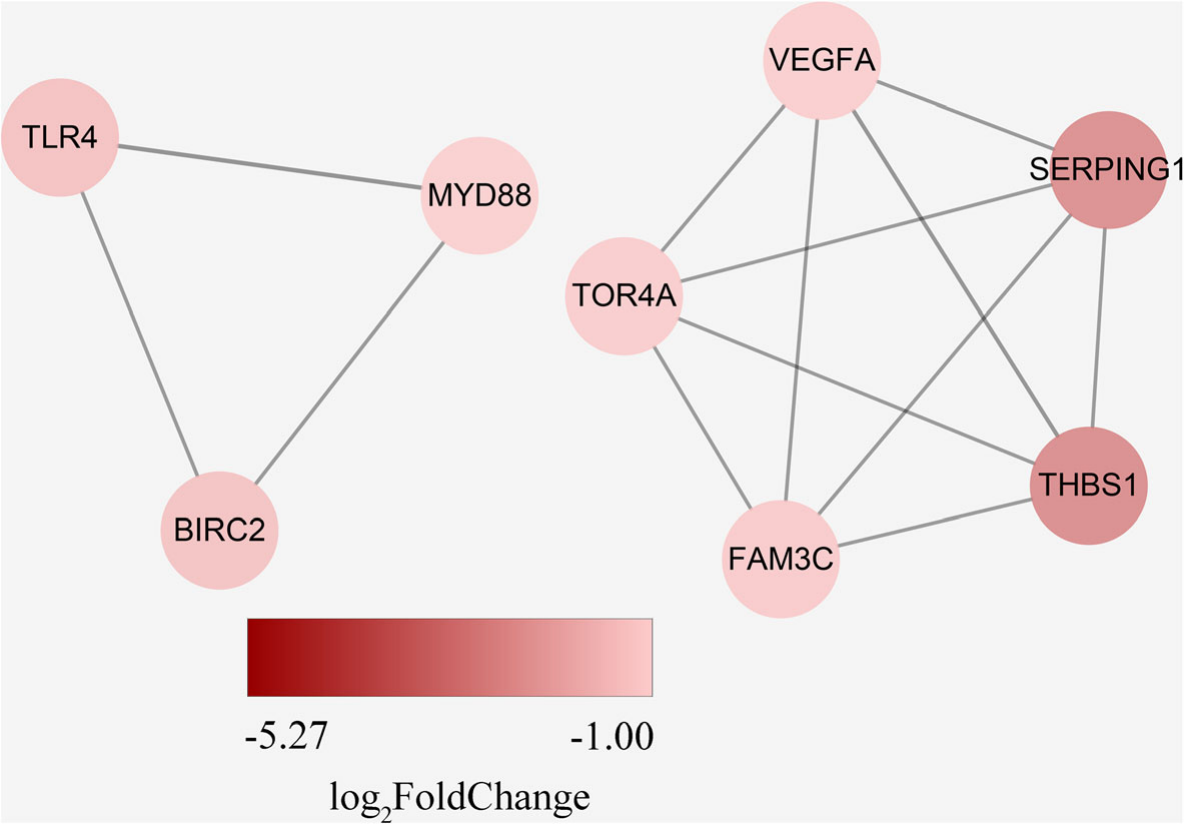
Modules of the protein-protein interaction network. The color of nodes indicates the degree of foldchange, the value was “-log_2_FoldChange”. The edge between proteins represent interactions between them and the confidence score were more than 0.7

The PPI networks for SDEGs consisted of 250 proteins and 341 pairs of PPIs (Additional file 9: Table S7). The hub proteins exceeded 8 interactions, including *VEGFA*, *MYD88*, *IL15*, *SDC4*, *ITGB4*, *ITGB3*, *ITGB5*, *THBS1*, *JUN*, *k60*, *LAPR1*, *PLCG1*, *MAPK11*, *RHOC*, and *RHOJ* (Additional file 10: Fig. S3). Only two proteins exceeded 13 interactions, *ITGB3* and *VEGFA*, of which the *VEGFA* has 18 interactions. A total of 21 modules including 81 proteins were obtained using default criteria, in which 19 modules with node > 3 and a MCODE score > 3 (Additional file 11: Fig. S4). Among them, we found module 19th had the *MYD88*, *TLR4* and *BIRC2* genes, of which the *MYD88* and *TLR4* were enriched in the Toll-like receptor signaling pathway. The *VEGFA* gene were in module second and enriched in the cytokine-cytokine receptor interaction pathway (Fig. 5).

### Validation of gene expression by quantitative real-time PCR (qRT-PCR)

**Fig. 6:**
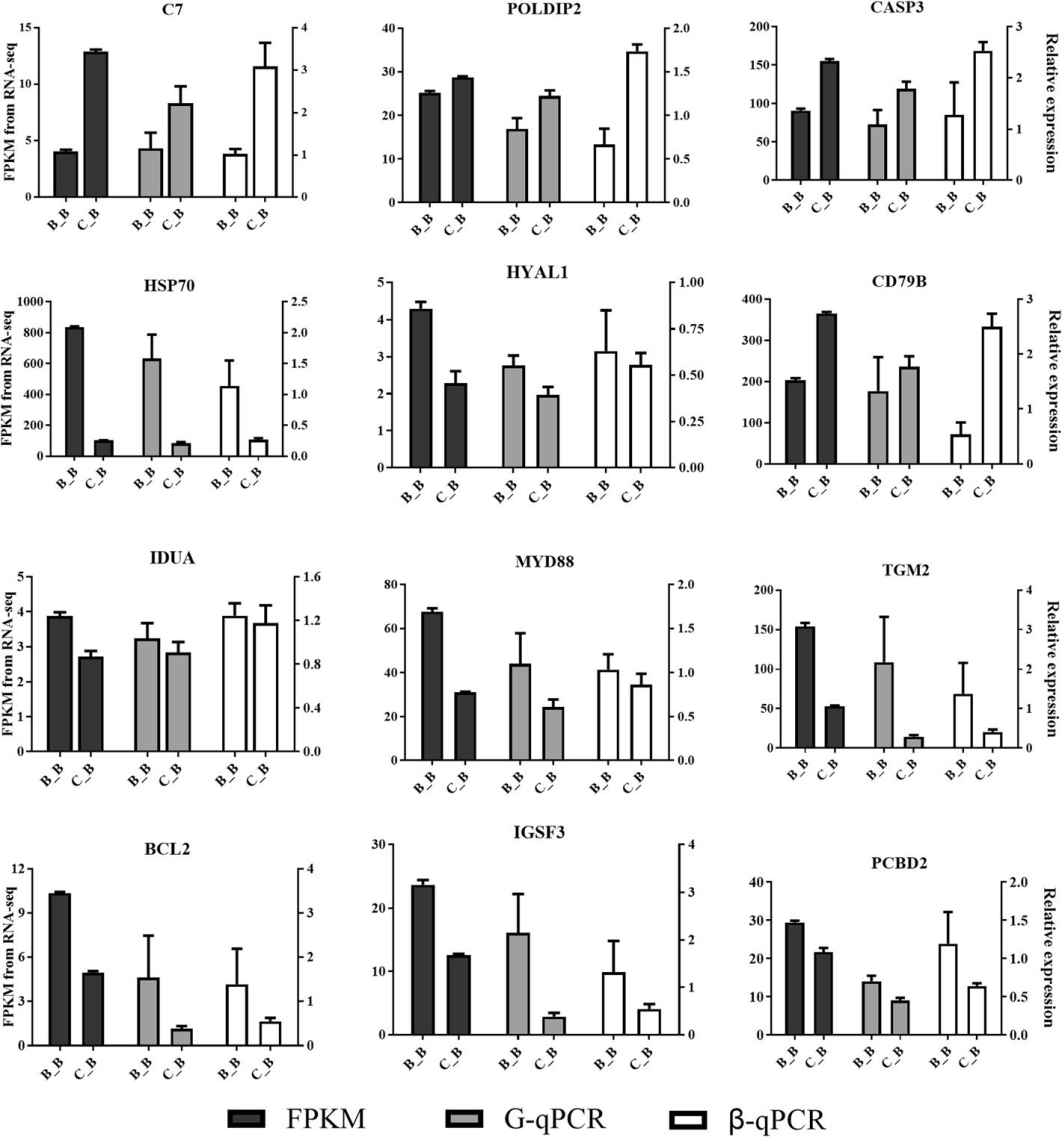
Compared the gene expression levels of RNA-seq with qRT-PCR. The left axis represents the expression levels determined by RNA-seq in FPKM units, and the right axis represents gene expression levels determined by qRT-PCR. Bars represent the mean (±SE) of three samples. The black column indicates the FPKM value; the grey column indicates the qRT-PCR used *GAPDH* as reference gene; the white column indicates the the qRT-PCR used *β-action* as reference gene

To confirm the accuracy of the differential expression analysis results, qRT-PCR was performed to validate the 12 randomly selected DEGs, including *C7*, *POLDIP2*, *CASP3* and *CD79B*, from the upregulated genes and *HSP70*, *IDUA*, *MYD88*, *TGM2*, *BCL2*, *PCBD2*, *HYAL1* and *IGSF3* from the downregulated genes, with *GAPDH* and *β-action* as the reference gene respectively. The qRT-PCR samples were the same as the RNA-seq samples, with three biological repeats for each group and three repetitions for each sample. The qRT-PCR results, with *GAPDH* and *β-action* as the reference genes, were consistent with the RNA-seq results (Fig. 6).

## Discussion

Glucocorticoid is a major stress hormone which increase rapidly in response to stress, such as CORT in bird [28]. Glucocorticoids regulate their effects by binding and activating glucocorticoid receptor (GR). Glucocorticoid receptor is a ligand-activated transcription factor, and has a zinc-finger motif DNA-binding domain, which can be used to regulate transcription both positively (transactivation) and negatively (trans-repression) [29]. In transactivation, GR activates downstream gene transcription by binding to a glucocorticoid response element (GRE). In trans-repression, GR interacts with other transcription factors, such as activator protein-1 (*AP-1*) and nuclear factor-kappa B (*NF-κB*), and indirectly inhibits transcription by reverse inhibition [30, 31]. Many of the GRs of the target genes are involved in regulating immune system and inflammatory response. The trans-repression activity of GR is the major basis for the anti-inflammatory and immunosuppressive effects of glucocorticoid. Previous research reported that feeding an experimental diet with 30 mg of added CORT/kg basal diet increased the plasma concentrations of CORT in chicken, indicating successful induction of stress response [16]. Therefore, we established a stress model by adding CORT to the diet at the same dose to analyze the molecular mechanism of stress affecting immune function [16].

In this study, we construct the stress model of chicken with administration of adding 30 mg CORT per kilogram of the basal diets referring to previous studies [16]. The aim of this study was to analysis molecular regulation mechanisms of the stress influencing immune function. Hence, the stress model was evaluated by detecting the indicators related to stress, including the physiological indexes such as CORT, glucose (GLU) and total protein (TP) in peripheral blood of chickens, serum CD3^+^, CD4^+^, IgG, TNF-α, IL-1β, IL-6 and relative lymphoid organ weights. The results showed that the levels of serum CORT, GLU, TP, TNF-α, IL-1β and IL-6 were increased significantly, the levels of alkaline phosphatase (ALP), CD3^+^ and CD4^+^ were decreased significantly (Additional files 12 and 13: Tables S8 and S9), however the relative bursa of Fabricius weights were no significant difference (results not shown). Corticosterone is a major stress hormone which increase rapidly in response to stress [32]. The higher serum CORT level, the more obvious the stress. The increase of serum GLU level after stress may be related to the induction of CORT. Corticosterone could reduce the utilization of GLU in various tissues of the body and enhance the hepatic gluconeogenesis. Moreover, CORT could promote protein metabolism and increases blood protein concentration. Cytokines are important mediators between the neuroendocrine system and the immune system. Stress could stimulate the release of inflammatory factors such as TNF-α, IL-1β, IL-6, and cause inflammatory response. Stress also could decrease the number of T cells and reduce cellular immunity function [33]. Our results indicated that the stress model of chicken was successfully constructed.

The bursa of Fabricius is the unique central immune organ of birds and play a central role in the immune response process [27]. B lymphocytes of birds are derived from bone marrow pluripotent stem cells and migrated to bursa of Fabricius, then in which differentiated and maturated. There are not only B lymphocytes in the bursa of Fabricius, but also many other constituent cells and tissues. They provide suitable microenvironment for differentiation and maturation of B lymphocytes. Exporting enough B cells from the bursa of Fabricius is essential for maintaining a normal peripheral B cell population and potential humoral immune responses [34]. Hence, the bursa of Fabricius as entire experimental sample was selected in this study. Indentifying genes and pathways from all cells in the bursa of Fabricius that are involved in immunomodulation in stress model can provide targets for understanding the molecular mechanisms of stress affecting immune function. Considering the influence of the heterogeneity in cell composition of the bursa of Fabricius to the interpretation of the data, the expression and function of candidate genes would be comprehensively analyzed through the annotations of database and immunohistochemical location in our subsequent studies.

Cathelicidin-B1-like (*CATHB1*, also known as *CATHL1*) was the most highly expressed gene in the bursa of Fabricius of the C_B group and the fourth highest expressed gene of B_B group. *CATHB1* is one of the four cathelicidin genes encoded by the chicken genome to form a family of vertebrate-specific immune molecules produced mainly by the bursa of Fabricius[35]and showed preferential expression in bursa of Fabricius [36]. Cathelicidin genes encode host defense proteins that have both antimicrobial activity and immunomodulatory functions [37]. Diverse functions that found in mammalian cathelicidins including chemotaxis of inflammatory and immune cells, stimulation of phagocytosis and activation and differentiation of immune cells [38]. Since it is unclear in which cell the chicken cathelicidin genes are expressed, the induction of local gene expression or an influx of cathelicidin expressing cells will increase the expression of cathelicidin genes [39], the highly expression of *CATHB1* need further researched.

Inflammation is one of the major reactions of the immune system against infection and irritation. Chronic inflammation is related to the expression of chemokines, cytokines and adhesion molecules. Pro-inflammatory cytokines, such as *TNF-α*, *IL-1*, *IL-8*, *IFN-γ* (interferon gamma) and *IL-11*, play an important role in mediating chronic inflammation. In our research, most of the cytokines in the CORT-treated group were significantly downregulated DEGs, such as *IL-1 β* (log_2_FC = − 1.426), *IL-18* (log_2_FC = − 1.3586), *IL-7* (log_2_FC = − 1.3558) and so on. Glucocorticoid has been shown to downregulate a variety of pro-inflammatory cytokines, including *IL-1 β*, *IL-2*, *IL-4*, *IL-6*, *IL-8*, *IL-11*, *IL-12*, *GM-CSF* and *TNF-α*, whereas glucocorticoid upregulated the expression of some anti-inflammatory genes including *IL-1 R2*(decoy receptor), *CC10*, *IL-1 receptor antagonist* and *Lipocortin-1* [40]. Of these downregulated genes, the *IL-6* gene was the only gene that contains a GRE promoter and recruit GR when glucocorticoids were present [41]. In the tethering mechanism, GR and transcription factors such as *NF-kB* and *AP-1* were directly interact; thus GR can be recruited to genomic regulatory regions and can increase or decrease the transcription of genes that contain no GRE site via transactivation or trans-repression, respectively [42]. Therefore, the tethering mechanism may play an important role in the downregulation of genes that contain no GRE sites, such as *IL-1 β* and *IL-18*, when an organism is treated with CORT.

According to the results of GO term, KEGG pathway enrichment and PPI network analysis of the 1434 SDEGs, *VEGFA* and *ILs* (such as *IL15*) via the cytokine-cytokine receptor interaction pathway, *MYD88* and *TLR4* via the Toll-like receptor signaling pathway may play important roles in the regulation of immune function caused by stress. *VEGF* is among the known tumor-associated factors that inhibits immune cell function. It inhibits T-cell development and may contribute to tumor-induced immune suppression [43]. *ILs* play an important role in mediating inflammation. In this study, there was no direct interaction relationship between *VEGF* and *IL15*, which severally play an immunoregulatory role through their upstream and downstream genes. Toll-like receptor play pivotal roles in the innate immune defense mechanism [44]. *MyD88* is the most common adaptor protein in the Toll-like receptor pathways. All TLRs use *MyD88* alone or in combination with other adapters, except *TLR3* [45]. The expression of *TLR4*-*MyD88* in B1a cells is critical for the IgM-dependent atheroprotection that reduces the CD4^+^ and CD8^+^ T cells infiltrates and enhances the *TGF-β1* expression while also reducing the lesion inflammatory cytokines *TNF-α*, *IL-1 β*, and *IL-18* [46]. Astragalus polysaccharides (APS) can regulate host immune response by activating the *TLR4*/*MYD88* signaling pathway [47]. The seizure-induced *TLR4*-mediated *MyD88*-dependent signaling pathway plays a key role in activating microglia and triggering neuron apoptosis [48]. *MyD88 and TLR4 genes* were significantly downregulated in our research which may indicate that it is regulated by CORT to change the immune function of the host via the *TLR4*/*MYD88* signaling pathway.

Further research for the molecular mechanisms of stress influence immune function is still necessary to reach the goal of increased disease resistance in stressed chicken. Determining the functional impacts of *MYD88* knockdown or *TLR4* overexpression on B cells will verity the importance of Toll-like receptor signaling pathway. Characterization of the effects of CORT on B-cells survival and proliferation and expression of *VEGFA* gene would be needed to further verify our experimental result and whether they have a positive or negative effect on immune capabilities. To characterize the interaction of stress and immune function, we need further investigation the expression and functional changes in cell proliferation and migration.

## Conclusion

In summary, we constructed and evaluated the stress model of chicken with administration of adding 30 mg CORT per kilogram of the basal diets. A total of 1434 SDEGs were identified in bursa of Fabricius of chicken using RNA-seq technology. Through GO enrichment, KEGG pathway and PPI network analysis, *VEGFA*, *ILs* (such as *IL15*) via the cytokine-cytokine receptor interaction pathway, *MYD88* and *TLR4* via the Toll-like receptor signaling pathway may play important roles in the regulation of immune function under stress condition with CORT administration. The results provide a basis for uncovering the molecular regulation mechanism of stress-influenced immune function in poultry.

## Materials and methods

### Experimental animals and tissue samples

Sixty healthy Gushi cocks of similar weight and 7 days of age were obtained from the Animal Center of Henan Agricultural University, randomly assigned to two groups, and held in a clean and relatively isolated room. All cock feeding and management were conducted in accordance with the chick feeding and management manual but without routine immunization and beak trimming in the feeding process. Control group (B_B) was fed the basal diet only, while the experimental group (C_B) was fed a basal diet, with the addition of 30 mg CORT/kg of basal diet [49](SIGMA -ALDRICH, SHANGHAI, CHINA, Purity ≥92%). Three cocks of each group were randomly selected and euthanized by exsanguination after 7 days of the dietary treatment. The stress model was evaluated by detecting the indicators related to immune/stress. The statistical analyses were performed with Graphpad Prism 5 (Graphpad Sofware, San Diego, CA), *P* ≤ 0.05 were considered statistically significant. The data are presented as the means ± SEM. Bursa of Fabricius tissues were harvested quickly and stored at − 80 °C until use.

### RNA extraction

The total RNA was isolated from the bursa of Fabricius tissues of chickens using an RNAiso Plus kit (Takara, Kyoto, Japan). Total RNA was purified using a TruSeq RNASample Prep Kit v2 (New England Biolabs, Ipswich, MA, USA). RNA degradation and contamination were detected using 1% agarose gels. The purity of the total RNA was assessed with a NanoPhotometer® spectrophotometer (IMPLEN, CA, USA). The concentration of the total RNA was measured using a Qubit® RNA Assay Kit in Qubit® 2.0 Fluorometer (Life Technologies, CA, USA). The integrity of the total RNA was estimated using an RNA Nano 6000 Assay Kit of the Bioanalyzer 2100 system (Agilent Technologies, CA, USA).

### Library construction and transcriptome sequencing

Six mRNA sequence libraries were constructed: one for every sample (B_B_1, B_B_2, B_B_3 and C_B_1, C_B_2, C_B_3). A total of 3 μg RNA per sample was needed to generate the sequencing libraries using NEBNext® Ultra™ RNA Library Prep Kit for Illumina® (NEB, Ipswich, MA, USA), following the manufacturer’s protocol and adding index codes to attribute sequences to each sample. Briefly, mRNA with a polyA tail was purified from total RNA using poly-T oligo-attached magnetic beads by the combination of A-T. The mRNA was fragmented and the cDNA was synthesized and purified with AMPure XP system (Beckman Coulter, Beverly, USA) to allow preferential selection of cDNA fragments of 250~300 bp in length. The cDNA library was enriched by PCR with Phusion High-Fidelity DNA polymerase, Universal PCR primers, and Index (X) Primer. At last, the PCR products were purified (AMPure XP system), and the library quality was assessed on the Agilent Bioanalyzer 2100 system and Q-PCR. Index-coded cDNA fragment libraries were analyzed using a paired-end 2 × 150 nt Illumina HiSeq platform controlled by data collection software. The image data were outputted and transformed into raw data and stored in FASTQ (fq) format after sequencing. The Q20, Q30, and GC content of the clean data were calculated at the same time. Raw sequencing data were processed through Perl scripts to remove reads containing the adapter, reads containing more than 10% poly-N, and reads of low quality (reads containing more than 50% bases with Q_phred_ ≤ 20). All downstream analyses were based on the resulting clean data.

### Mapping and transcript abundance estimation

The chicken genome assembly (*Gallus Gallus* 4.0) and gene model annotation files were downloaded from Ensemble. Bowtie (version: 2.2.3) [50] was used to build the index of the reference genome, and TopHat (version: 2.1.0) [51] was used to perform genome mapping on the clean data. TopHat can generate a database of splice junctions based on the gene model annotation file, thus allowing the alignment of reads that contain gaps compared with the genome and consummating catalogs of alternative splicing events. Based on the alignment results, the sequencing data of each library were assembled separately by Cufflinks (version 2.1.1) [52]. Cufflinks were used to assemble the gene annotation files, compare them with the original gene annotation files, determine the genes that are not included in the original annotations and optimize the gene’s position, thereby supplementing and modifying the original annotation file. The transcripts were mapped to the reference annotation files using the Cuffcompare, a program in the Cufflinks package, to sort out novel genes; discover new exon regions of known genes; and optimize the starting and ending positions of known genes [53]. Then, HTSeq (version: 0.6.1) [54] was used to count the number of fragments for each gene. The FPKM (fragments per kilobase of exon model per million mapped reads) considers the effect of the sequencing depth and gene length to standardize the read count for the comparison between the different genes and samples through the use of a Perl script (FPKM = total exon fragments/mapped reads in millions × exon length in kb) [55].

### Identification and functional annotation of DEGs

Differentially expressed genes of two groups (three biological replicates per group) were performed using the DESeq2 R package (version:1.14.1) by mean normalised read-counts [56]. DESeq2 provides statistical routines for determining DEGs using a model based on the negative binomial distribution. The input count values must be raw counts of sequencing reads for DESeq’s statistical model to hold, as only the actual counts allow assessing the measurement precision correctly [56, 57]. So that we did no filter of FPKM value before the DESeq analyses. The Benjamini and Hochberg’s approach were used to control the FDR of the *P*-value. We identified DEGs as genes with an adjusted P-value (padj) < 0.05 and FPKM > 1, and SDEGs as genes with padj < 0.05, |log_2_FC| ≥ 1, and FPKM > 1. Cluster analysis was used to cluster genes with the same or similar expression patterns, which might have similar functions or participate in the same biological process. Cluster analysis of a heat-map for SDEGs was performed by the pheatmap R package.

The GOseq R package [58] was used to identify significantly enriched GO terms with corrected *P*-values < 0.05 by SDEGs. The KEGG (http://www.genome.jp/kegg/) pathway is the major public pathway-related database for understanding the biological functions of genes, especially large-scale molecular datasets generated by genome sequencing and other high-through put experimental technologies. We used KOBAS software to test the statistical enrichment of SDEGs in KEGG pathways [59] to identify enriched pathways and clarify the group differences in cellular pathways. Pathways with corrected P-value (q-value) < 0.05 were identified as significantly enriched by the SDEGs.

### The protein-protein interaction analysis of DEGs

The PPI network of the SDEGs was analyzed using the interaction relation in the STRING protein interaction database (http://string-db.org, Organism: Chicken) by extracting the SDEGs list from the database pertaining to chickens and selected the interaction relation with confidence score > 0.7. The PPI networks were visualized by Cytoscape (version 3.6.1). Based on the scale-free property of interaction networks, hub proteins were found by counting the number of interactions of each network node has with other nodes [60]. Network module analysis was performed through MCODE in Cytoscape with default parameters (Degree Cutoff: 2, Node Score Cutoff: 0.2, K-Core: 2, Max. Depth: 100).

### qRT-PCR

We randomly selected 12 genes (including 4 upregulated genes and 8 downregulated genes) for qRT-PCR in the two groups to confirm the reproducibility and accuracy of the RNA-Seq gene expression data. Using an RNAiso Plus kit (Takara, Kyoto, Japan), total RNA was isolated from bursa of Fabricius tissues of chickens. After the check of RNA quality, as described in the section on RNA extraction, reverse transcription was performed using the PrimeScript™ RT Reagent Kit with gDNA Eraser (Takara, Kyoto, Japan) according to the manufacturer’s protocol.

The qRT-PCR experiments were performed using a LightCycler® 96 Real-Time PCR system (Roche Applied Science) in a 25-μL reaction volume containing 12.5 μL of 2 × SYBR® Premix Ex TaqTM II (Tli RNaseH Plus) (Takara, Kyoto, Japan), 1.25 μL each of the forward and reverse primers (10 μM), 8 μL of deionized water, and 2 μL (approximately 100 ng) of cDNA. The *GAPDH* gene was used as the reference gene, and the primers of 12 genes were designed using the Primer-BLAST website of NCBI (https://www.ncbi.nlm.nih.gov/tools/primer-blast/). The primers are shown in the Additional file 14: Table S10. The thermal cycling conditions were 3 min at 95 °C, followed by 37 reaction cycles (95 °C for 30 s, 60 °C for 30 s, and 72 °C for 30 s), and an extension for 10 min at 72 °C. We calculated the relative gene expression levels with the comparative CT method (also referred to as the 2^-△△CT^ method) [61], with three replicates each reaction. The data are presented as the means ± SEM.

## Additional files

Additional file 1: **Table S1.** Characteristics of the reads from bursa of Fabricius libraries obtained from 2 groups. ^1^Multiple mapped = number of clean reads and the ratio that matched two or more positions in the genome. ^2^Uniquely mapped = number of clean reads and the ratio that matched only one position in the genome. (DOCX 17 kb)

Additional file 2: **Figure S1.** Pearson correlation between samples. (TIF 362 kb)

Additional file 3: **Figure S2.** Venn diagram of global genes expressed in two groups. Red indicates the genes specific expressed in experimental group, blue indicates the genes specific expressed in the control group and purple indicates the genes expressed in both group. (TIF 504 kb)

Additional file 4: **Table S2.** The gene expression level of the two groups. Sheet 1 showed the top 10 highest expression genes in two groups. Sheet 2 showed the FPKM and read count of all genes. Sheet 3 showed the mean normalised read-counts by DESeq2. (XLSX 3969 kb)

Additional file 5: **Table S3.** The DEGs in the C_B group compared with the B_B group. Sheet 1 showed the SDEGs, sheet 2 showed the downregulated SDEGs (padj < 0.05, |log_2_FC| ≥ 1, and FPKM > 1), sheet 3 showed the upregulated SDEGs, sheet 4 showed the DEGs (padj < 0.05, FPKM > 1). (XLSX 1358 kb)

Additional file 6: **Table S4.** The GO enrichment of SDEGs of the C_B group and B_B group. Sheet 1 showed the top 30 most significantly enriched GO terms and sheet 2 showed all the enriched GO terms. (XLSX 974 kb)

Additional file 7: **Table S5.** The KEGG enrichments of the differentially expressed genes of the C_B group and B_B group. Sheet 1 showed the top 20 most significantly enriched KEGG pathways and sheet 2 showed all the enriched KEGG pathways. (XLSX 47 kb)

Additional file 8: **Table S6.** The correlation coefficient between receptor genes expression and ligand genes expression in the cytokine-cytokine receptor. (XLSX 21 kb)

Additional file 9: **Table S7.** Protein-protein interaction network for all SDEGs. Sheet 1 showed the interactions with a confidence score > 0.7, sheet 2 showed the log_2_FoldChange values of genes in sheet1, sheet 3 showed the KEGG analysis result of the genes in the 19 modules. (XLSX 29 kb)

Additional file 10: **Figure S3.** Protein-protein interaction network for all SDEGs. The color of nodes indicates the degree of foldchange, the value was “-log_2_FoldChange”. Red color of label indicates proteins which exceeded 8 interactions with others, while black color indicates proteins which below 8 interactions. The edge between proteins represent interactions between them and the confidence score were more than 0.7. (TIF 695 kb)

Additional file 11: **Figure S4.** 19 modules of the protein-protein interaction network with node > 3 and a MCODE score > 3. The color of nodes indicates the degree of foldchange, the value was “-log_2_FoldChange”. The edge between proteins represent interactions between them and the confidence score were more than 0.7. (TIF 617 kb)

Additional file 12: **Table S8.** Effects of CORT treatment on the serum biochemical indexes of chickens. Data are shown as the mean ± SE. Different lowercase letters (a and b) in same column indicate significant differences among the C_B and B_B groups (P < 0.05). (DOCX 13 kb)

Additional file 13: **Table S9.** Effects of CORT treatment on immune related indexes of chickens. Data are shown as the mean ± SE. Different lowercase letters (a and b) in same column indicate significant differences among the C_B and B_B groups (P < 0.05). (DOCX 15 kb)

Additional file 14: **Table S10.** List of the genes and primers used for qRT-PCR validation. (XLSX 12 kb)

## Abbreviations

ACTH: Adrenocorticotropic hormone
AP-1: Activator protein-1
B2M: *β*-2-microglobulin
CATHB1: Cathelicidin-B1-like
CORT: Corticosterone
CRH: Corticotropin-releasing hormone
GR: Glucocorticoid receptor
GRE: Glucocorticoid response element
IGLL1: Immunoglobulin lambda-like polypeptide 1
MR: Mineralocorticoid receptor
MYD88: Myeloid differentiation primary-response gene 88
NF-Κb: Nuclear factor-kappa B
RSAD2: Radical S-adenosyl methionine domain-containing 2
VEGFA: Vascular endothelial growth factor
