# Supplementary material for: Transcriptome profile in bursa of Fabricius reveals potential mode for stress-influenced immune function in chicken stress model
Source: BMC Genomics. 2018 Dec 13;19:918. doi: 10.1186/s12864-018-5333-2 (PMC6293626; doi:10.1186/s12864-018-5333-2)
Supplement: Supplementary file 1 — Table S1. Characteristics of the reads from bursa of Fabricius libraries obtained from 2 groups. 1Multiple mapped = number of clean reads and the ratio that matched two or more positions in the genome. 2Uniquely mapped = number of clean reads and the ratio that matched only one position in the genome. (DOCX 17 kb) [file 12864_2018_5333_MOESM1_ESM.docx]

| **Table S1. Characteristics of the reads from bursa of Fabricius libraries obtained from 2 groups** | | | | | | | | | |
| --- | --- | --- | --- | --- | --- | --- | --- | --- | --- |
|  |  |  |  |  |  |  |  |  |  |
| **Sample name** | **Raw reads** | **Clean reads** | **Total mapped** | **Multiple mapped**^1^ | **Uniquely mapped**^2^ | **Exon%** | **Intron%** | **Intergenic**% | **GC content (%)** |
| B_B_1 | 56,268,498 | 54,504,710 | 46,449,889 (85.22%) | 1035469 (1.90%) | 45,414,420 (83.32%) | 68.4 | 8.8 | 22.8 | 49.70 |
| B_B_2 | 54,929,184 | 52,040,158 | 44,383,971 (85.29%) | 958245 (1.84%) | 43,425,726 (83.45%) | 68.5 | 8.6 | 22.9 | 48.95 |
| B_B_3 | 48,920,052 | 47,474,890 | 41,100,201 (86.57%) | 897014 (1.89%) | 40,203,187 (84.68%) | 68.1 | 9.0 | 22.9 | 48.33 |
| C_B_1 | 60,324,068 | 59,020,642 | 51,107,845 (86.59%) | 1114634 (1.89%) | 49,993,211 (84.7%) | 71.5 | 8.3 | 20.2 | 49.06 |
| C_B_2 | 62,611,916 | 59,032,044 | 51,205,764 (86.74%) | 1108305 (1.88%) | 50,097,459 (84.86%) | 72.0 | 8.5 | 19.5 | 48.43 |
| C_B_3 | 60,156,136 | 57,301,570 | 49,818,693 (86.94%) | 1089700 (1.90%) | 48,728,993 (85.04%) | 72.0 | 8.7 | 19.3 | 48.52 |

^1^Multiple mapped = number of clean reads and the ratio that matched two or more positions in the genome.

^2^Uniquely mapped = number of clean reads and the ratio that matched only one position in the genome.
