# Supplementary figures and images for: Transcriptome profile in bursa of Fabricius reveals potential mode for stress-influenced immune function in chicken stress model
Source: BMC Genomics. 2018 Dec 13;19:918. doi: 10.1186/s12864-018-5333-2 (PMC6293626; doi:10.1186/s12864-018-5333-2)

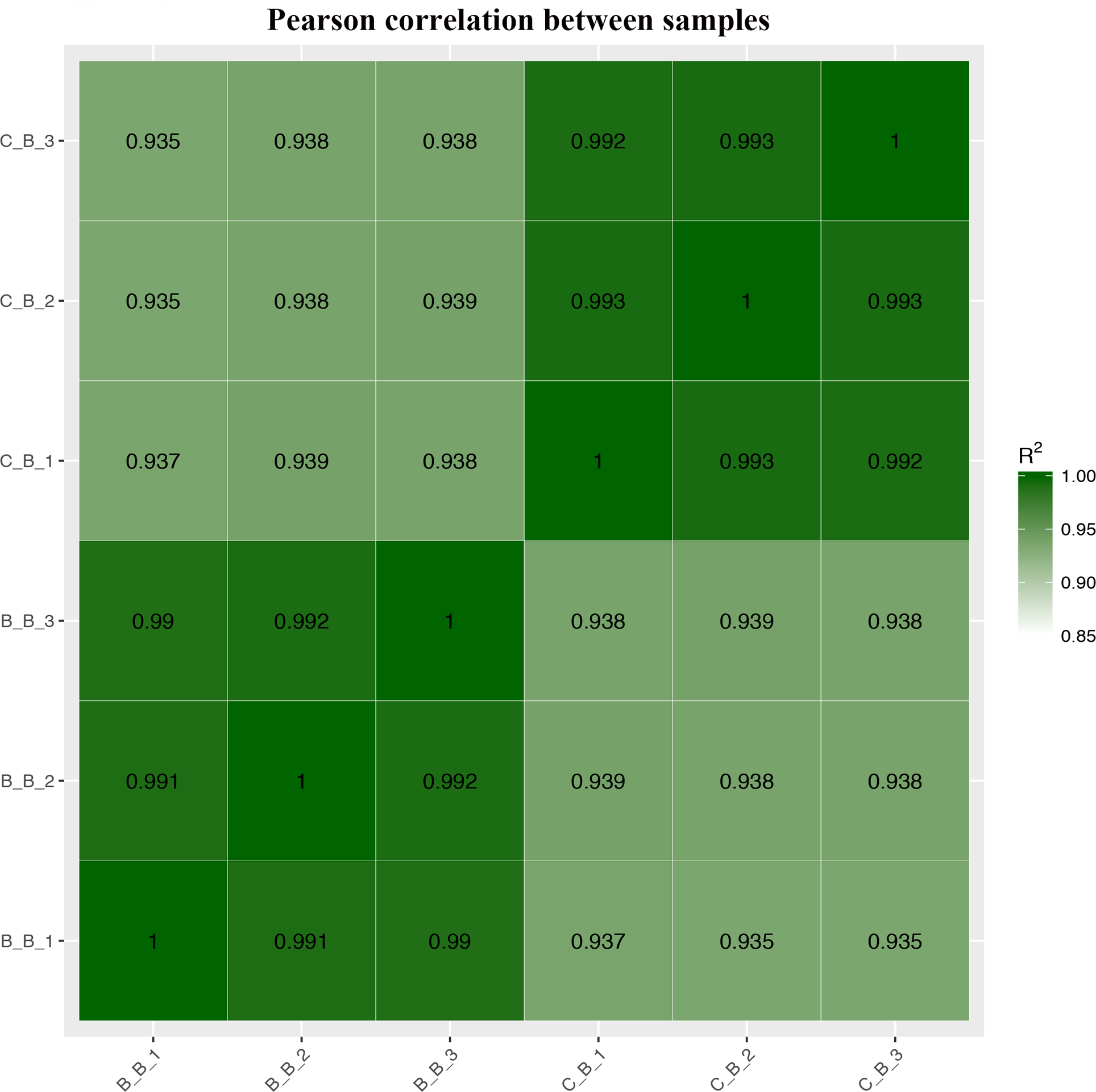

Supplement: Supplementary file 2 — Figure S1. Pearson correlation between samples. (TIF 362 kb) [file 12864_2018_5333_MOESM2_ESM.tif]

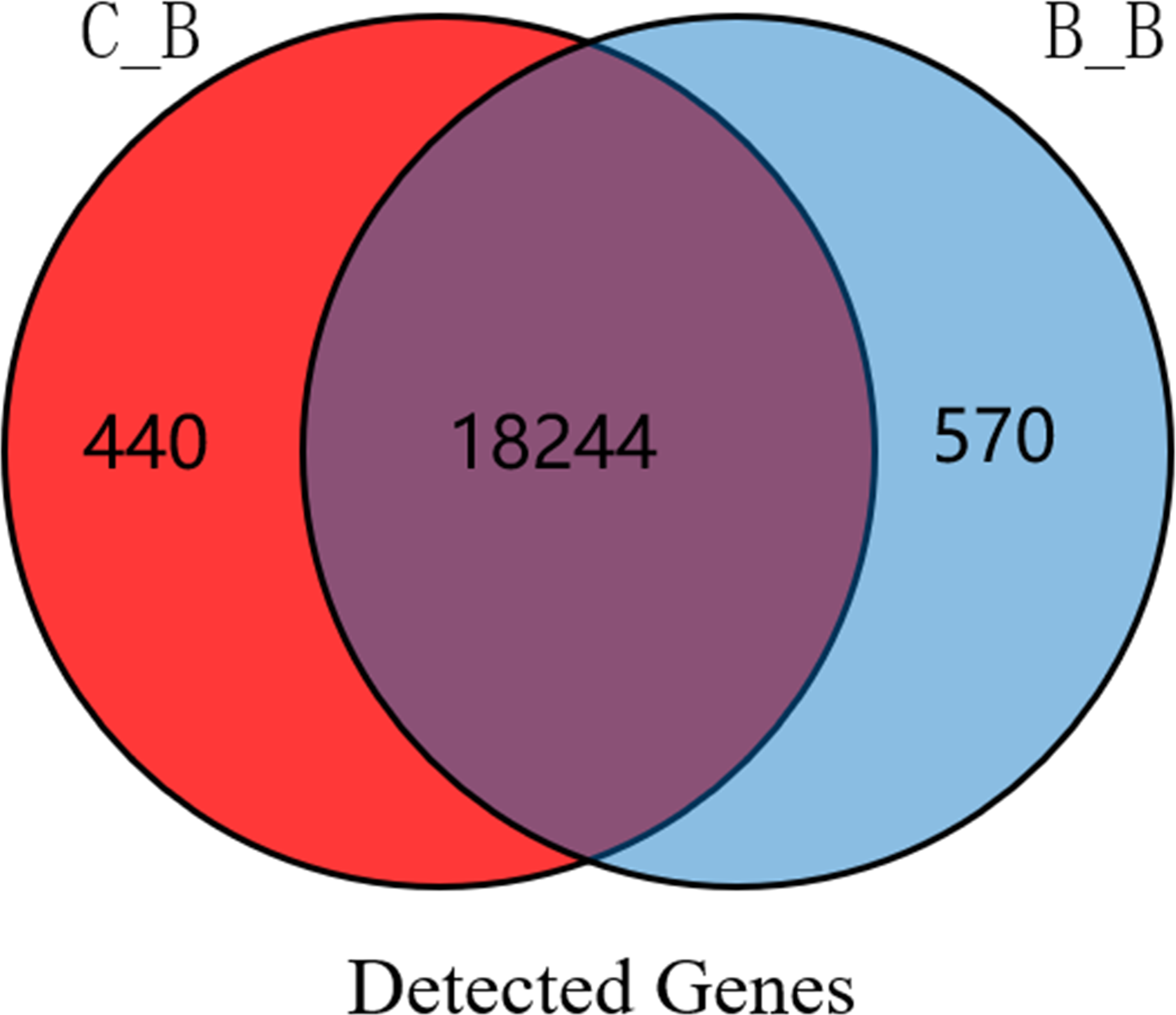

Supplement: Supplementary file 3 — Figure S2. Venn diagram of global genes expressed in two groups. Red indicates the genes specific expressed in experimental group, blue indicates the genes specific expressed in the control group and purple indicates the genes expressed in both group. (TIF 504 kb) [file 12864_2018_5333_MOESM3_ESM.tif]

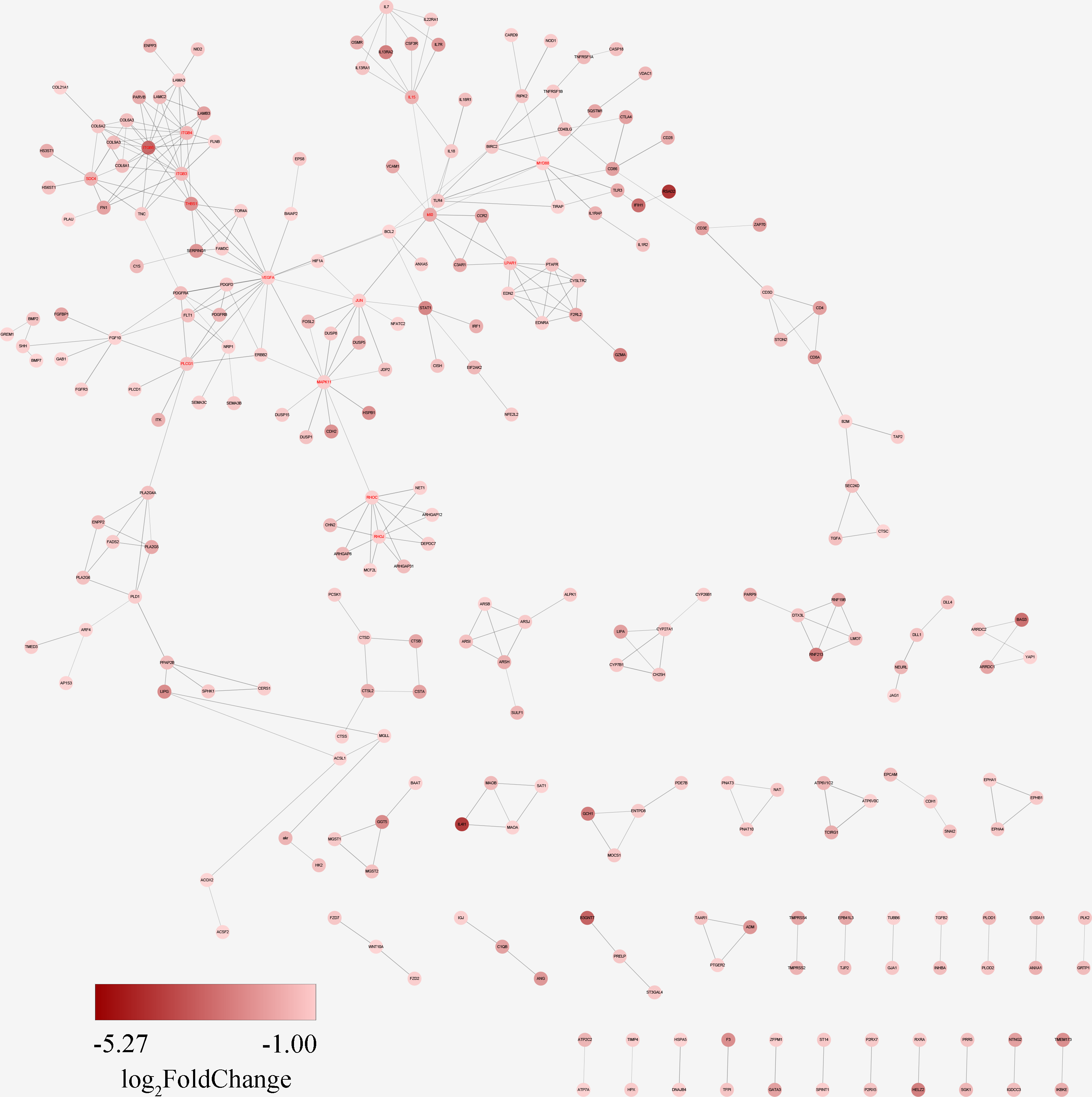

Supplement: Supplementary file 10 — Figure S3. Protein-protein interaction network for all SDEGs. The color of nodes indicates the degree of foldchange, the value was “-log2FoldChange”. Red color of label indicates proteins which exceeded 8 interactions with others, while black color indicates proteins which below 8 interactions. The edge between proteins represent interactions between them and the confidence score were more than 0.7. (TIF 695 kb) [file 12864_2018_5333_MOESM10_ESM.tif]

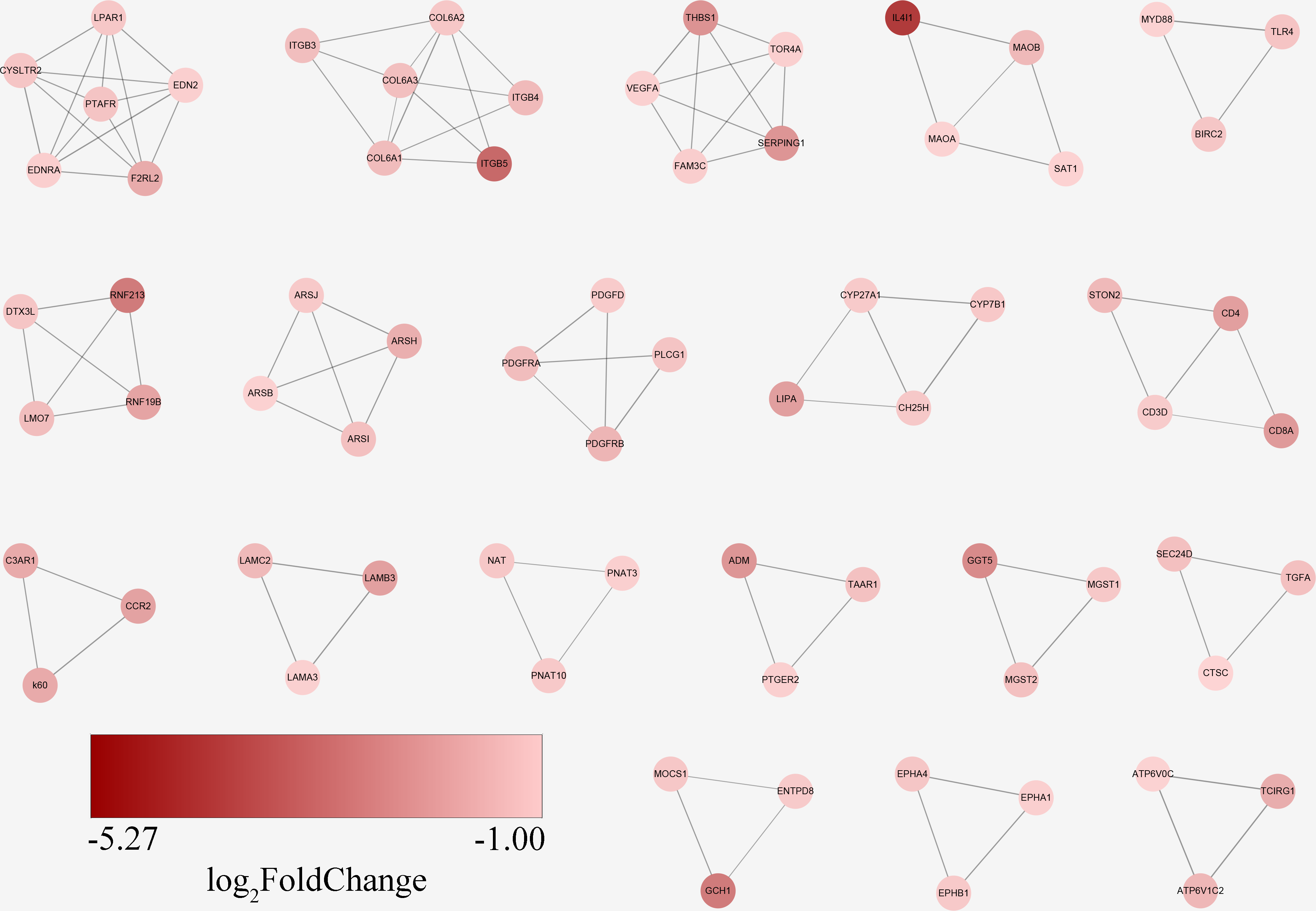

Supplement: Supplementary file 11 — Figure S4. 19 modules of the protein-protein interaction network with node > 3 and a MCODE score > 3. The color of nodes indicates the degree of foldchange, the value was “-log2FoldChange”. The edge between proteins represent interactions between them and the confidence score were more than 0.7. (TIF 617 kb) [file 12864_2018_5333_MOESM11_ESM.tif]
