# Supplementary material for: Transcriptome profile in bursa of Fabricius reveals potential mode for stress-influenced immune function in chicken stress model
Source: BMC Genomics. 2018 Dec 13;19:918. doi: 10.1186/s12864-018-5333-2 (PMC6293626; doi:10.1186/s12864-018-5333-2)
Supplement: Supplementary file 12 — Table S8. Effects of CORT treatment on the serum biochemical indexes of chickens. Data are shown as the mean ± SE. Different lowercase letters (a and b) in same column indicate significant differences among the C_B and B_B groups (P < 0.05). (DOCX 13 kb) [file 12864_2018_5333_MOESM12_ESM.docx]

**Table S8. Effects of CORT treatment on the serum biochemical indexes of chickens.** Data are shown as the mean ± SE. Different lowercase letters (a and b) in same column indicate significant differences among the C_B and B_B groups (P < 0.05).

| **Group name** | **CORT (nmol/L)** | **Glucose (mmol/L)** | **Triglycerides (mmol/L)** | **Total cholesterol (mmol/L)** | **Total protein**  **(g/L)** | | **Alkaline phosphatase (IU/L)** |
| --- | --- | --- | --- | --- | --- | --- | --- |
| B_B | 25.66±1.71^b^ | 14.73±0.94^b^ | 1.14±0.07 | 4.34±0.23 | 32.97±2.02^b^ | 1698±442.9^a^ | |
| C_B | 30.94±2.63^a^ | 16.27±0.36^a^ | 1.04±0.02 | 3.89±0.09 | 37.47±5.64^a^ | 924.6±19.8^b^ | |
