# Supplementary material for: Transcriptome profile in bursa of Fabricius reveals potential mode for stress-influenced immune function in chicken stress model
Source: BMC Genomics. 2018 Dec 13;19:918. doi: 10.1186/s12864-018-5333-2 (PMC6293626; doi:10.1186/s12864-018-5333-2)
Supplement: Supplementary file 13 — Table S9. Effects of CORT treatment on immune related indexes of chickens. Data are shown as the mean ± SE. Different lowercase letters (a and b) in same column indicate significant differences among the C_B and B_B groups (P < 0.05). (DOCX 15 kb) [file 12864_2018_5333_MOESM13_ESM.docx]

| **Table S9. Effects of CORT treatment on immune related indexes of chickens.** Data are shown as the mean ± SE. Different lowercase letters (a and b) in same column indicate significant differences among the C_B and B_B groups (P < 0.05). | | | | | |  |
| --- | --- | --- | --- | --- | --- | --- |
| **Group name** | **CD3+**  **(U/ml)** | **CD4+**  **(U/ml)** | **IgG**  **(ng/ml)** | **TNF-α**  **(ng/L)** | **IL-1β**  **(ng/L)** | **IL-6**  **(ng/L)** |
| B_B | 16.03±1.68^a^ | 4.58±0.25^a^ | 678.61±67.58 | 148.81±13.75^b^ | 21.88±2.91^b^ | 34.16±6.80^b^ |
| C_B | 14.24±0.79^b^ | 3.43±0.39^b^ | 665.24±92.40 | 167.12±9.73^a^ | 24.03±1.95^a^ | 40.18±2.27^a^ |
